# Supplementary material for: Utilizing river and wastewater as a SARS-CoV-2 surveillance tool in settings with limited formal sewage systems
Source: Nat Commun. 2023 Nov 30;14:7883. doi: 10.1038/s41467-023-43047-y (PMC10689440; doi:10.1038/s41467-023-43047-y)
Supplement: Supplementary file 1 — Supplementary Information [file 41467_2023_43047_MOESM1_ESM.pdf]

Supplementary Figures

Figure S1 Additional collection and site information A) Topography of the city of Blantyre with wastewater collection sites in black. B) the 25 'highly' informative sites that are being used for future studies defined by our cluster and hotspot analysis C) The region-specific dot plot shows 22 areas of Blantyre sampled during phase 1 (2020) and/or phase 2 (2021-2022) by negative (blue) or positive (red) for SARS-CoV-2. This also shows there was over sampling and under sampling of some regions of the city and regions with higher SARS-CoV-2 detection. D) SARS-CoV-2 positive samples compared to monthly cumulative rain data. Maroon circles are positive samples and light pink are negative samples. Green bar graphs are the monthly rain data for Blantyre based on CHIRPS rainfall estimates[1,2] from the Climate Hazards Center – NASA, USAID, NOAA. Although Beta and Omicron correspond to the raining season Delta falls within the dry season.

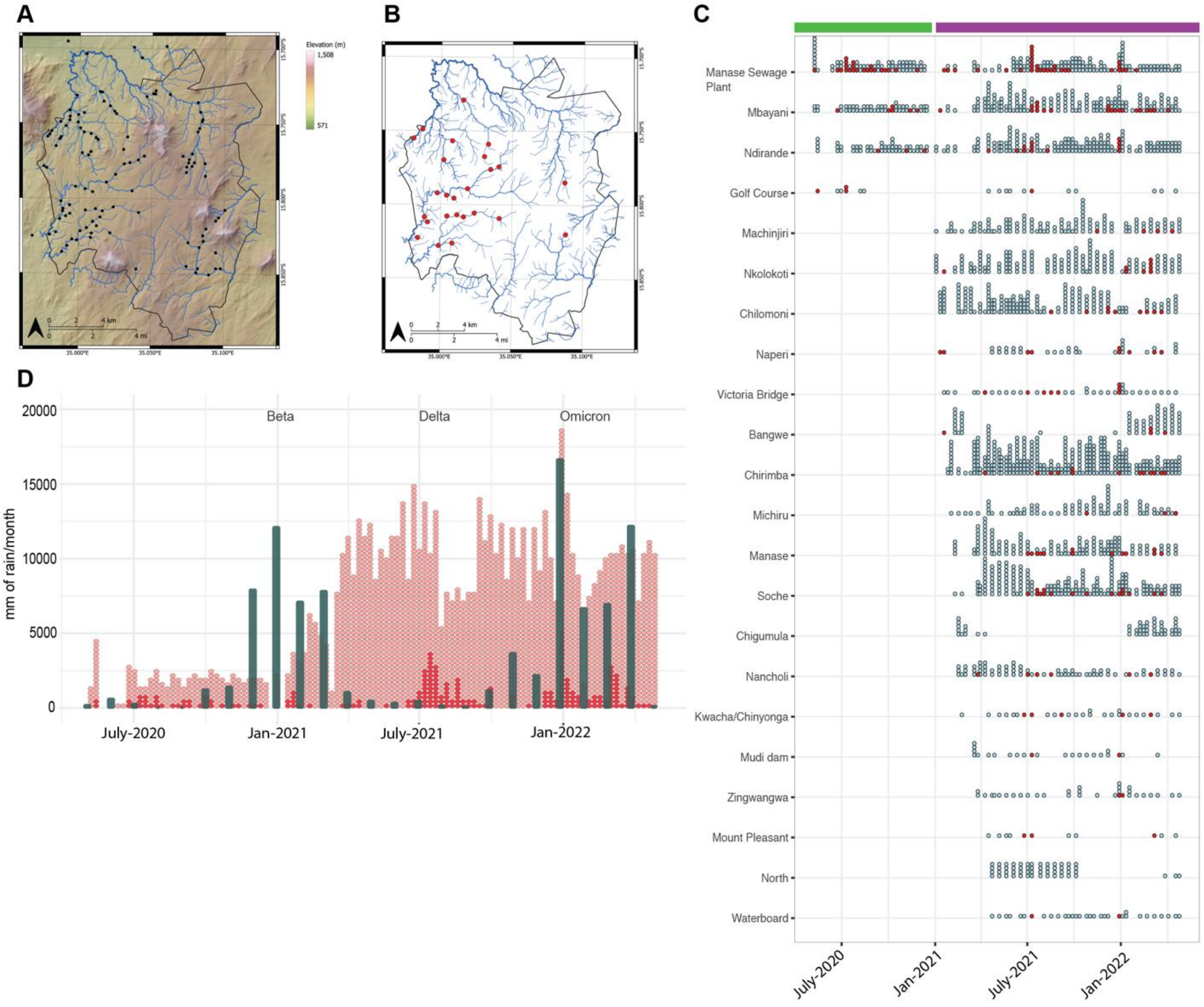

Figure S2. Full collection RT-PCR data. 30ml grab concentrated with 10% PEG and tested for SARS-CoV-2 using the CDC N1 RT-PCR assay over time (x-axis) and cycle threshold (y-axis) show the low viral loads of these samples. Phase 1 is in light blue, and Phase 2 is in dark blue. The genomic copies per liter range from 1150copies (Ct=39) to 140735copies (Ct=30.3).

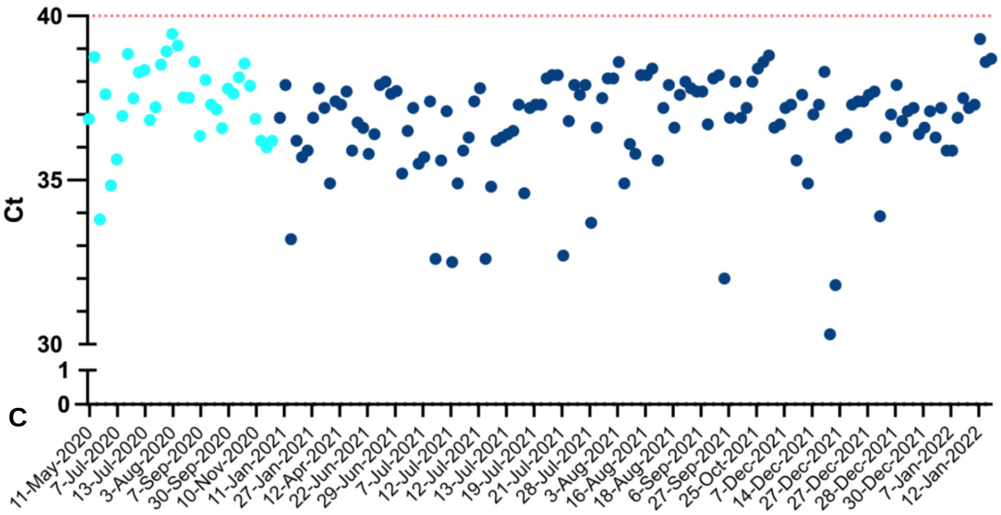

Figure S3. Estimates of lag time between ES and passive case (DHO) surveillance. Yellow represents the rolling average of ES positivity over time compared to passive case surveillance numbers (bar graph in gray) where the y-axis is total on number of positive cases per day. DHO data is over a fold higher in numbers than the active case data (Fig2), but low numbers of Delta compared to high numbers of Omicron could not be independently validated and may represent reporting issues. Spline analysis of both ES (red) and passive surveillance (black) show how closely linked peaks in both detection methods are over multiple waves.

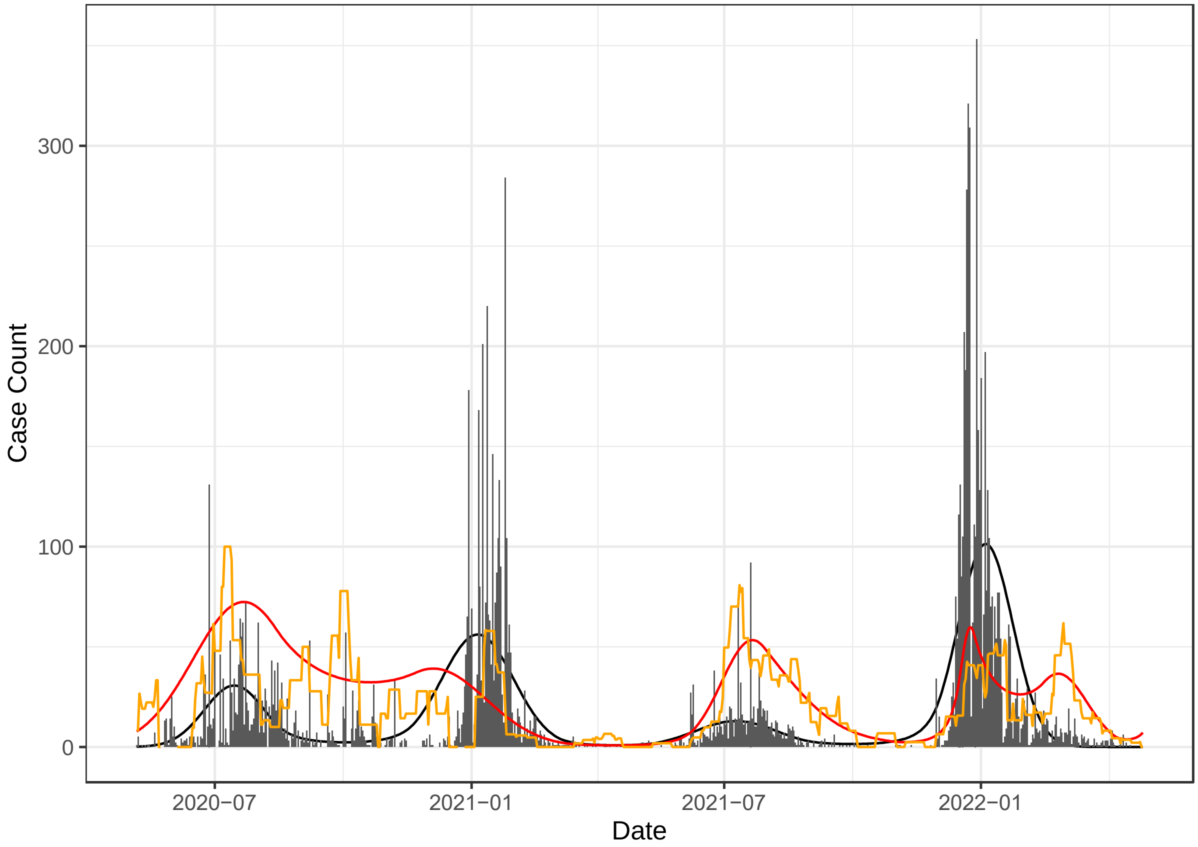

Figure S4: 1,000 realizations from the multivariate normal distribution parameterized by the ES model, showing uncertainty in modeled ES detection rates.

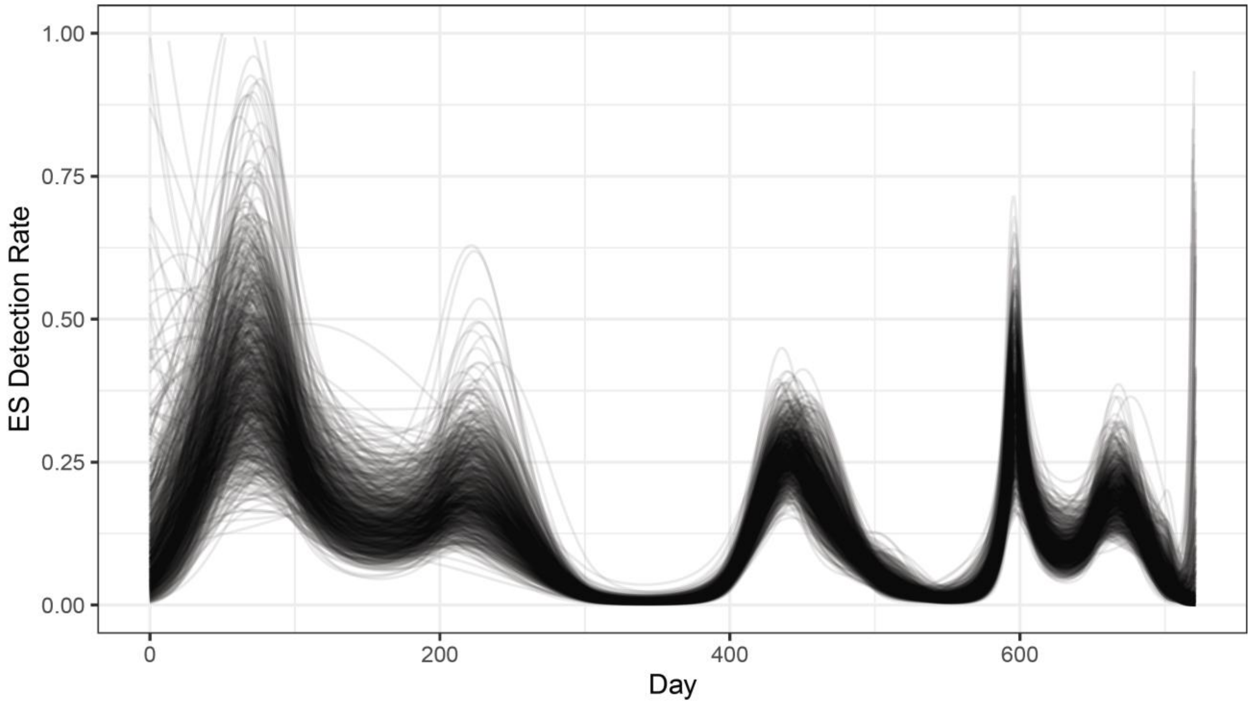

Figure S5. Clinical prevalence rates for each wave are shown as black points. ES detection rates over time are shown in red, and temporally adjusted to show the best-fit estimate of the lag (left panel). Residual deviance by lag for each wave, showing the tested range of lagged effects and best-fit estimate (right panel).

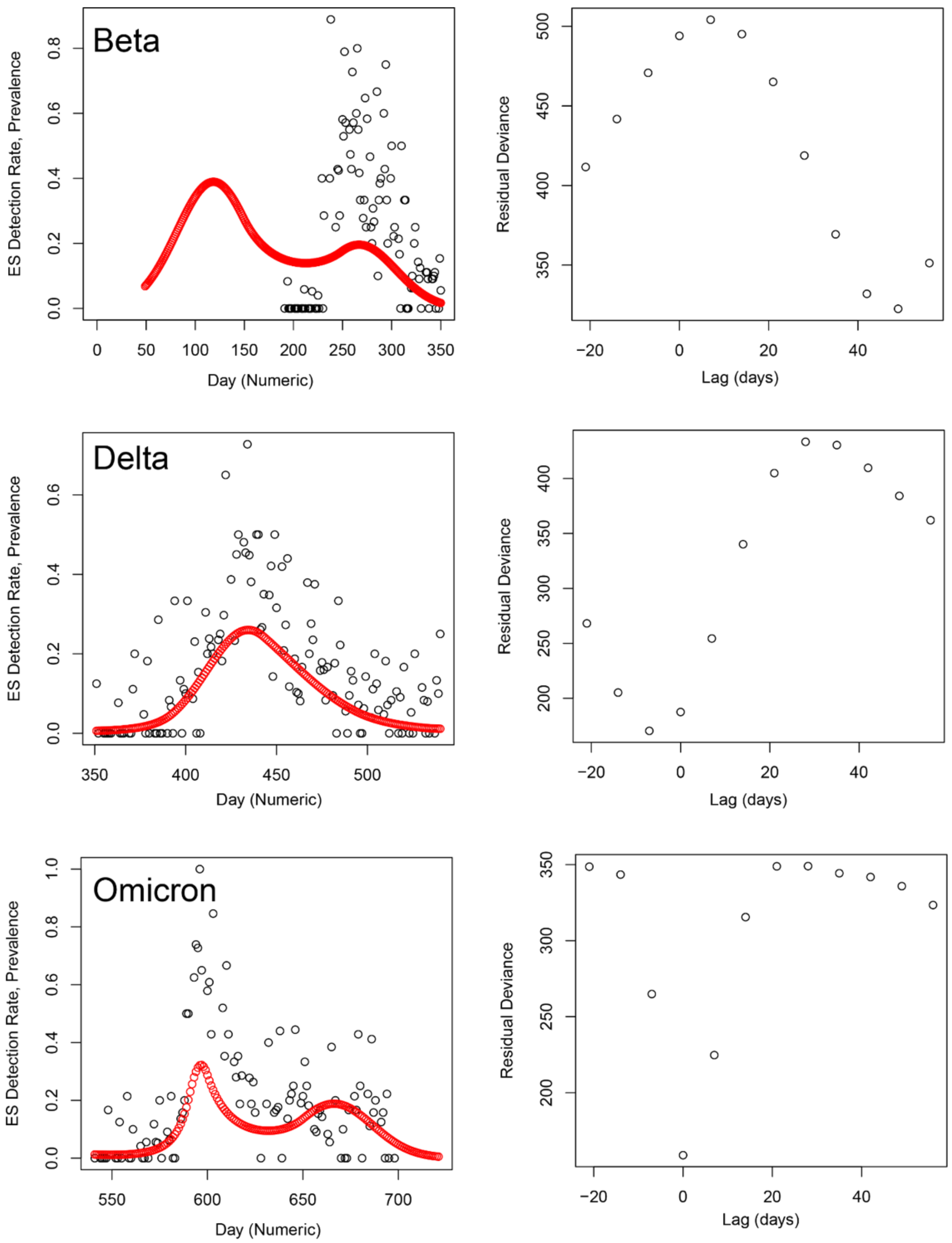

Figure S6. Predicted prevalence across ES detection rates for each modeled wave, where waves 1, 2, and 3 represent Beta (red), Delta (green), and Omicron (blue), respectively.

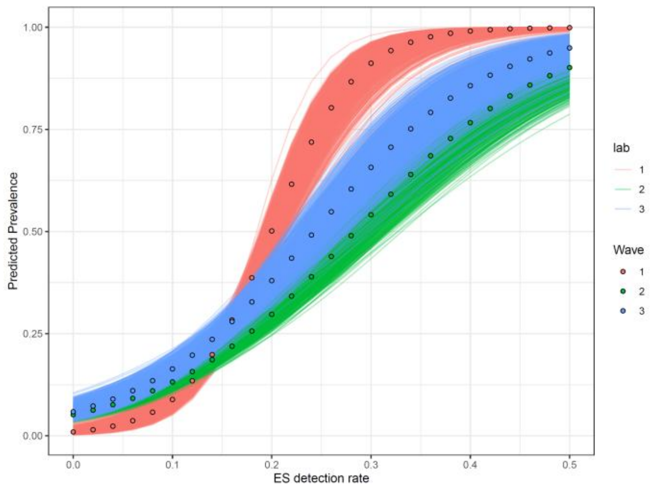

Figure S7: Histogram of the p-values for ES waves 1-3, where waves 1, 2, and 3 represent Beta, Delta, and Omicron, respectively.

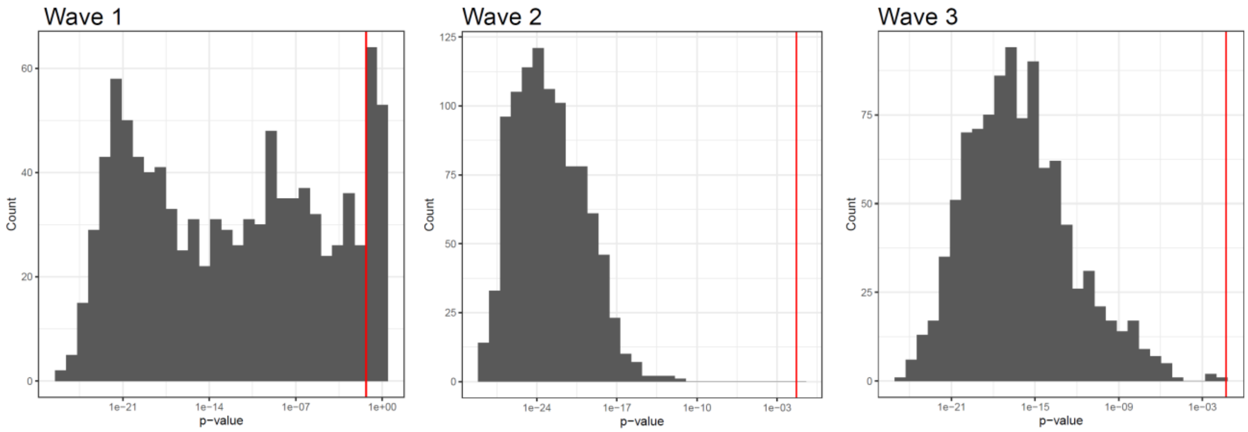

Figure S8. Sequencing Methods

A) Percentage genome coverage (y-axis) for ARTIC+Minion method vs EasySeq+Illumina method for all samples B) Percentage genome coverage (y-axis) for ARTIC+Minion (blue) method vs EasySeq+Illumina method (red) per matched sample

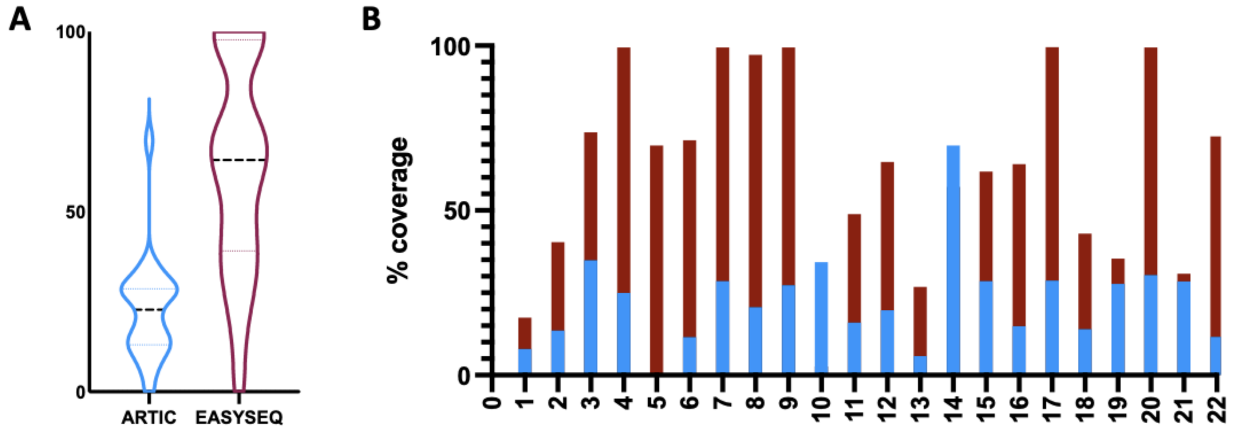

Figure S9: heatmap mutations: Omicron SNPs show putative early detection of the VOC. The heatmap shows all Omicron SNPs on the y-axis (blue=BA.1-specific, red=BA.2-specific, green=BA.1/2 shared mutation) and individual ES samples overtime on the x-axis where months are by color. In September (samples have some key Omicron SNPs but lack the full repertoire of SNPs which become dominant by December.

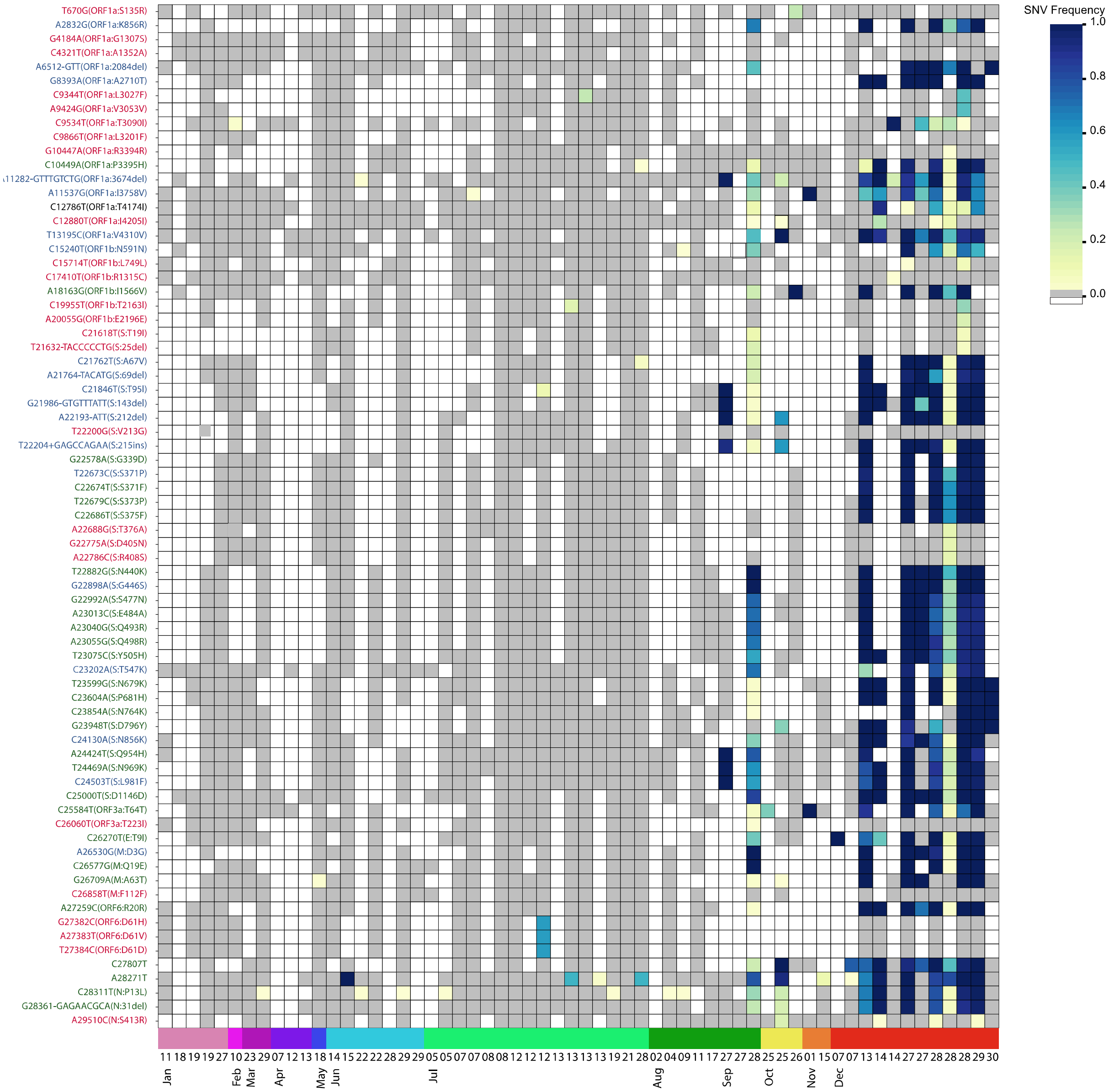

Figure S10. Deeper analysis of Sept 2021 genome with possible contamination A) Detailed analysis of Sept-2021 Omicron signatures in one sample with matching mutations to a patient (EPI_ISL11246699) shown in green while non matching SNPs are shown in red. B) is the same sample sequenced from the original wastewater does not show this contamination but contains a small number of BA.2/BA.4 mutations (white squares indicate no sequencing depth at that site). The re-sequenced sample contained key Omicron SNPs including specific to BA.2 and BA.4, which is not in line with the putative early Omicron signatures.

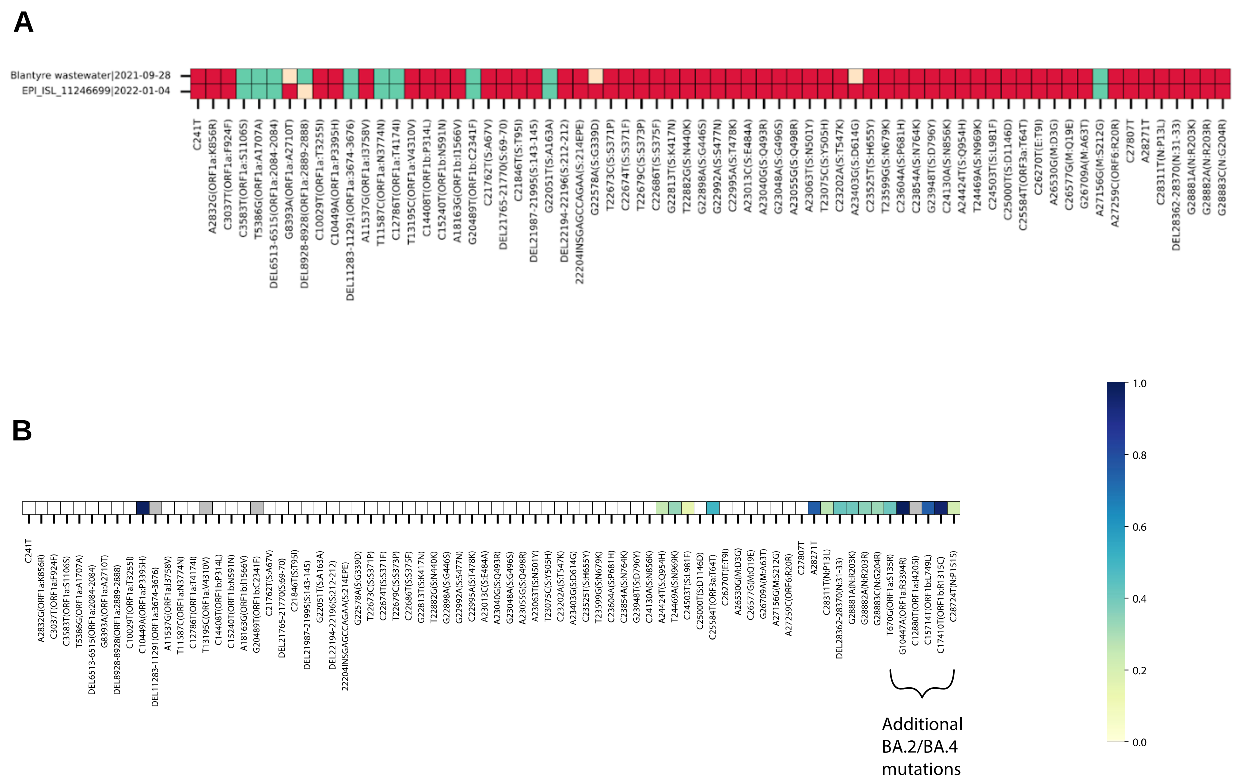

Figure S11: Phylogenetic dating analyses: Time-calibrated Bayesian phylogenetic analysis of the early Omicron sample under three considerations: genome assembly with only physically linked mutations (green), genome assembly with the inclusion of the frequency-linked BA.1.14 mutation (orange) and all frequency-linked mutations (blue). Since Omicron was identified as a minority component in the mixture, our initial consensus approach may incorrectly include additional mutations associated with Delta lineages at a comparable frequency. Hence, we considered the inclusion of two additional cases, corresponding to using only physically linked mutations and only physically linked mutations plus the frequency-linked C12786T (Orf1a:T4174I) mutation (a lineage-defining mutation for BA.1.14, which later circulated in Malawi).

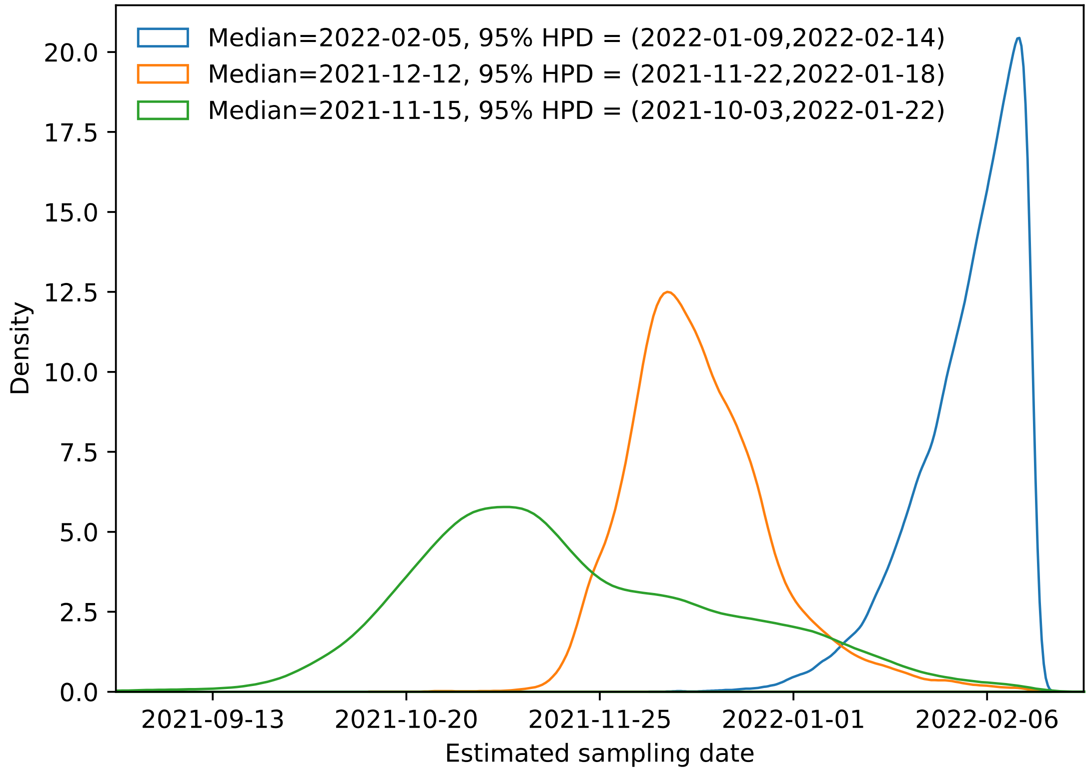

Figure S12. Maximum likelihood phylogenetic tree for Delta variant show ES samples from Malawi (in blue) cluster mainly with patient samples from Malawi South Africa and Kenya

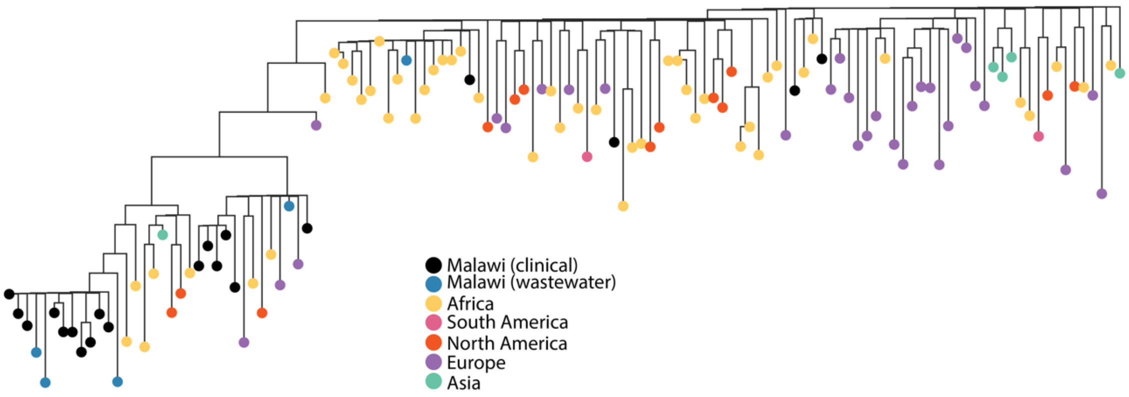

Figure S13. Laboratory methods comparison We spikes wastewater samples with known genomic copies of SARS-CoV-2 to compare concentration methods A) Comparison of filter vs unfiltered grab known positive samples showed no significant difference between methods B) comparison of wastewater samples spiked with $2x10^8$ SARS-CoV-2 copies and then concentrated with PEG (yellow) and MF (blue) showed modest but insignificant improvement for detection by PEG for one sample but overall, both methods are comparable in our setting. This also showed we could recover about half of the SARS-CoV-2 genomic copies ($1x10^8$).

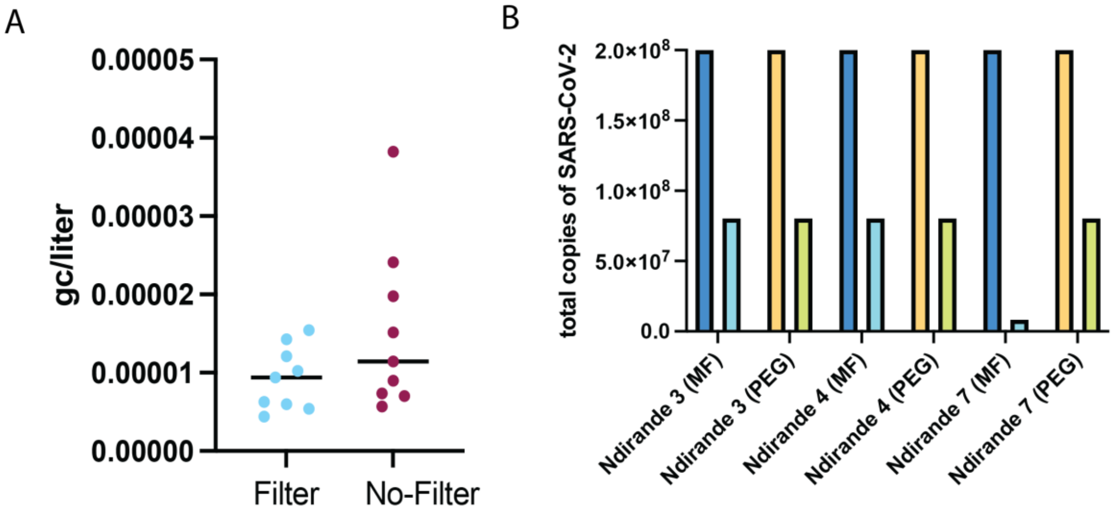

Supplementary Table

Table S1 – RT-PCR primers, probe and gblock are based on the CDC N1 assay. The gblock was generated in January 2020 and this area of remains conserved but SNPs may have changed with each new VOC.

>CCDC-N-F<br><br>GGGGAACTTCTCCTGCTAGAAT
>CCDC-N-P<br><br>TTGCTGCTGCTTGACAGATT
>CCDC-N-R<br><br>CAGACATTTTGCTCTCAAGCTG
>CCDC-N-gblock-Broad-Design<br><br>GAAATTAATACGACTCACTATAGGGTGACCCTGTGGGTTTTACACTTAAAAACACAGTCTGTACCGTCTGCGGTATGTGGAAAGGTTATGGCTGTAG<br>TTGTGATCAACTCCGCGAACCCATGCTTCAGTCAGCTGATGCACAATCGTTTTGAGTGAAATGGTCATGTGTGGCGGTTCACTATATGTTAAACCAG<br>GTGGAACCTCATCAGGAGATGCCACAACTGCTTATGCTAATAGTGTTTTTAACATTTGTCAGAGACAGGTACGTTAATAGTTAATAGCGTACTTCTT<br>TTTCTTGCTTTCGTGGTATTCTTGCTAGTTACACTAGCCATCCTTACTGCGCTTCGATTGTGTGCGTACTGCTGCAATATTGTTGATAATGGACCCCA<br>AAATCAGCGAAATGCACCCCGCATTACGTTTGGTGGACCCTCAGATTCAACTGGCAGTAACCAGAATGGAGAACGCAGTGGGGCGCGATCAAAACAA<br>CGTCGGCCCCAAGGTTTACCCAATAATACTGCGTCTTGGTTCACCGCTCTCACTCAACATGGCAAGGAAGACCTTAAATTCCCTCGAGGACAAGGCGT<br>TCCAATTAACACCAATAGCAGTCCAGATGACCAAATTGGCTACTACCGAAGAGCTACCAGACGAATTCGTGGTGGTGACGGTAAAATGAAAGATCTC<br>AGTCCAAGATGGTATTTCTACTACCTAGGAACTGGGCCAGAAGCTGGACTTCCCTATGGTGCTAACAAAGACGGCATCATATGGGTTGCAACTGAGG<br>GAGCCTTGAATACACCAAAAGATCACATTGGCACCCGCAATCCTGCTAACAATGCTGCAATCGTGCTACAACTTCCTCAAGGAACAACATTGCCAAA<br>AGGCTTCTACGCAGAAGGGAGCAGAGGCGGCAGTCAAGCCTCTTCTCGTTCCTCATCACGTAGTCGCAACAGTTCAAGAAATTCAACTCCAGGCAGCA<br>GTAGGGGAACTTCTCCTGCTAGAATGGCTGGCAATGGCGGTGATGCTGCTCTTGCTTTGCTGCTGCTTGACAGATTGAACCAGCTTGAGAGCAAAAT<br>GTCTGGTAAAGGCCAACAACAACAAGGCCAAACTGTCACTAAGAAATCTGCTGCTGAGGCTTCTAAGAAGCCTCGGCAAAAACGTACTGCCACTAAA<br>GCATACAATGTAACACAAGCTTTCGGCAGACGTGGTCCAGAACAAACCCAAGGAAATTTTGGGGACCAGGAACTAATCAGACAAGGAACTGATTACA<br>AACATTGGCCGCAAATTGCACAATTTGCCCCCAGCGCTTCAGCGTTCTTCGGAATGTCGCGCATTGGCATGGAAGTCACACCTTCGGGAACGTGGTTG<br>ACCTACACAGGTGCCATCAAATTGGATGACAAAG

Viral detection in wastewater

V2_Feb2022

Prepared by Kayla Barnes

Supplementary Note 1

MATERIALS

- 70% EtOH
- Pipets (P2,P10,P200 & P1000)
- Tips (filter or non-filter)
- 50 ml Falcon
- Cold transportation box
- PEG 8000
- NaCl (0.3M)
- Vortex/Centrifuge
- PBS - 1X
- 1.5ml Eppendorf's
- LunaScript RT
- Qiagen viral RNA extraction kit
- MicroAmp Optical 96 well Reaction Plate and Plate Seals pr plate specific to your machine
- qScript XLT One-Step RTqPCR ToughMix, Low ROX (95134)
- Enteric Virus Multiplex Panel Primer/Probe Mix
- Nuclease Free Water
- CDC positive control N1 SARSCov-2
- QuantStudio RTqPCR Machine or equivalent

Sample collection and storage

Grab sample with no filtration

Store 30-40mL samples in 50mL Flacon Conical tubes at -80 until processing

Ensure the sample is not overfilled before freezer

Sample concentration for viral extraction

1. Add 3g of PEG to the 30mL sample

2. Add 0.68g NaCl (0.3 M, Millipore Sigma) to the 30mL sample.

3. Centrifuge at 1200g*, 4°C** for 2 hours.
   * every centrifuge is different so anywhere from 800-2000g
   ** Centrifuge at CoM does not go to 4°. Room temp is OK but less ideal
   *** Can be shortened to 1 hour centifuge

a. Slowly poor out supernatant and discard- Take care to NOT REMOVE the pellet

b. To remove remaining supernatant use a P1000
c. It is OK to leave ≤100ul of supernatant

4. Add 100-300 uL sterile PBS to the pellet, and pipet up and down to combine the pellets for each sample
    a. Amount of PBS depends on how 'dirty' the sample is. If the pellet is larger and thick add more PBS
    b. Use the least amount of PBS possible

5. Vortex 2 minutes at maximum speed on vortex adapter to completely resuspend pellet.

6. Transfer sample to a (locking) 1.5 Eppendorph tube OR cryovial.

7. Proceed to extraction or Store at -80°C

    a. Note: storing at -80°C can decrease the detectable viral particles so whenever possible extract and proceed to PCR on the same day.

Manual Viral extraction – Qiagen viral RNA extraction kit

1. Extraction all samples plus a water control

2. Add MS2 or viral spike in (10,000 total copies) to each sample

3. Combine **500µl of Buffer AVL** to the **200µl ES-PBS** sample in the 1.5mL Eppendorf tube and mix by pulse-vortexing for 15s.

4. Incubate at room temperature (15–25°C) for 10 min.

5. Briefly centrifuge the tube to remove drops from the inside of the lid. Ensure that the lid is used on the centrifuge.

6. Add 500 µl of ethanol (molecular grade 96–100%) to the sample and mix by pulse-vortexing for 15s.

7. Briefly centrifuge the tube to remove drops from inside the lid. Ensure that the lid is used on the centrifuge.

8. Label the top of a spin column to keep track of the samples

9. Carefully apply 630µl of the solution from step 4 to the QIAamp Mini column without wetting the rim.

    Centrifuge at 6000 x g (8000 rpm) for 1 min
    place the column in a new collection tube

10. Carefully open the QIAamp Mini column, and repeat step 7.

Centrifuge at 6000 x g (8000 rpm) for 1 min
place the column in a new collection tube

11. Add 500 µl of Buffer AW1 to the column.

Centrifuge at 6000 x g (8000 rpm) for 1 min
place in a new collection tube

12. Add 500 µl of Buffer AW2 to the column.

Centrifuge at full speed (20,000 x g; 14,000 rpm) for 3 min.

13. To release the RNA from the column place the column in a clean, labelled 1.5 ml microcentrifuge tube add 50µl of Buffer AVE and incubate at room temperature for 1 min.

Centrifuge at 6000 x g (8000 rpm) for 1 min.

13. Save the remaining RNA in the same 1.5 Eppendorf tube at -80°C.

14. Proceed to COVID-19 RT-qPCR SOP or store RNA at -80°C for long term storage

---

Viral RT-qPCR

*You can purchase the CDC diagnostic assay from IDT or other suppliers

Primers

>CCDC-N-Forward<br><br>GGGGAACTTCTCCTGCTAGAAT
>CCDC-N-Probe<br><br>TTGCTGCTGCTTGACAGATT<br><br>*This can be any florescent your RT-PCR machine has. We use FAM
>CCDC-N-Reverse<br><br>CAGACATTTTGCTCTCAAGCTG
>CCDC-N-gblock-Broad-Design<br><br>GAAATTAATACGACTCACTATAGGGTGACCCTGTGGGTTTTACACTTAAAAACACAGTCTGTACCGTCTGCGGTATGTGGAAAGGTTATGGCTGTAG<br>TTGTGATCAACTCCGCGAACCCATGCTTCAGTCAGCTGATGCACAATCGTTTTGAGTGAAATGGTCATGTGTGGCGGTTCACTATATGTTAAACCAG<br>GTGGAACCTCATCAGGAGATGCCACAACTGCTTATGCTAATAGTGTTTTTAACATTTGTCAGAGACAGGTACGTTAATAGTTAATAGCGTACTTCTT<br>TTTCTTGCTTTCGTGGTATTCTTGCTAGTTACACTAGCCATCCTTACTGCGCTTCGATTGTGTGCGTACTGCTGCAATATTGTTGATAATGGACCCCA<br>AAATCAGCGAAATGCACCCCGCATTACGTTTGGTGGACCCTCAGATTCAACTGGCAGTAACCAGAATGGAGAACGCAGTGGGGCGCGATCAAAACAA<br>CGTCGGCCCCAAGGTTTACCCAATAATACTGCGTCTTGGTTCACCGCTCTCACTCAACATGGCAAGGAAGACCTTAAATTCCCTCGAGGACAAGGCGT<br>TCCAATTAACACCAATAGCAGTCCAGATGACCAAATTGGCTACTACCGAAGAGCTACCAGACGAATTCGTGGTGGTGACGGTAAAATGAAAGATCTC<br>AGTCCAAGATGGTATTTCTACTACCTAGGAACTGGGCCAGAAGCTGGACTTCCCTATGGTGCTAACAAAGACGGCATCATATGGGTTGCAACTGAGG<br>GAGCCTTGAATACACCAAAAGATCACATTGGCACCCGCAATCCTGCTAACAATGCTGCAATCGTGCTACAACTTCCTCAAGGAACAACATTGCCAAA<br>AGGCTTCTACGCAGAAGGGAGCAGAGGCGGCAGTCAAGCCTCTTCTCGTTCCTCATCACGTAGTCGCAACAGTTCAAGAAATTCAACTCCAGGCAGCA<br>GTAGGGGAACTTCTCCTGCTAGAATGGCTGGCAATGGCGGTGATGCTGCTCTTGCTTTGCTGCTGCTTGACAGATTGAACCAGCTTGAGAGCAAAAT<br>GTCTGGTAAAGGCCAACAACAACAAGGCCAAACTGTCACTAAGAAATCTGCTGCTGAGGCTTCTAAGAAGCCTCGGCAAAAACGTACTGCCACTAAA

GCATACAATGTAACACAAGCTTTCGGCAGACGTGGTCCAGAACAAACCCAAGGAAATTTTGGGGACCAGGAACTAATCAGACAAGGAACTGATTACA
AACATTGGCCGCAAATTGCACAATTTGCCCCCAGCGCTTCAGCGTTCTTCGGAATGTCGCGCATTGGCATGGAAGTCACACCTTCGGGAACGTGGTTG
ACCTACACAGGTGCCATCAAATTGGATGACAAAG

Mix primer and probes

1. Clean equipment and workspace with Rnase and 70% EtOH
2. Prepare the RT-PCR master mixes for N1 in a 1.5 Eppendorf tubes as follows:

samples = + 1 (positive) + 1 (NTC) + 2 (extra for pipetting error) =___________

Reagent	Volume each Reaction	N1
qScript XLT ToughMix	10 µL	
Primer/Probe mix	5 µL	
RNA sample	5.0 µL	
**TOTAL mastermix**	**15 µL**	

*You can reduce this by 50% if you are low on reagent but with ES samples best to use a full 5ul of RNA

1. Pipet 15ul of COVID master mix into each well taking care to pipet

2. Pipet 5ul of the RNA into each well

3. Thaw and pipette the single use positive serial dilution (10^1-10^5 copies/ul dilutions of the CDC positive control or gblock) and the Negative extraction control in H12

4. Seal plate, vortex for 10 seconds and spin plate to remove droplets and bubbles.

5. Use the COVID-19 RT-qPCR template on the Applied Bio 7500, Quantstudio or Viia

Check the parameters are correct based on :
Target = FAM, Cy5 and HEX Standard run
Passive reference = ROX

Thermocycling conditions are based on qScript mastermix

Step	Cycles	Temp	Time
RT	1	50°C	10 min
Enzyme activation	1	95°C	3 min
Amplification	45	95°C	3 sec
		55°C	30 sec

Positive sample is one which has a Ct <40

Supplementary File Citations

1 Dimitrova, A., McElroy, S., Levy, M., Gershunov, A. & Benmarhnia, T. Precipitation variability and risk of infectious disease in children under 5 years for 32 countries: a global analysis using Demographic and Health Survey data. *Lancet Planet Health* **6**, e147-e155 (2022). https://doi.org:10.1016/S2542-5196(21)00325-9

2 Funk, C. *et al.* The climate hazards infrared precipitation with stations--a new environmental record for monitoring extremes. *Sci Data* **2**, 150066 (2015). https://doi.org:10.1038/sdata.2015.66
