# Supplementary material for: Utilizing river and wastewater as a SARS-CoV-2 surveillance tool in settings with limited formal sewage systems
Source: Nat Commun. 2023 Nov 30;14:7883. doi: 10.1038/s41467-023-43047-y (PMC10689440; doi:10.1038/s41467-023-43047-y)
Supplement: Supplementary file 3 — Description of Additional Supplementary Files [file 41467_2023_43047_MOESM3_ESM.pdf]

Description of Additional Supplementary Files

File Name: Supplementary Data 1
Description: All information by site. All site names, areas, site type, location and collection numbers positivity, and if the site is considered highly informative

File Name: Supplementary Data 2
Description: Full dataset. Includes dates as both DD/MM/YY and MM/DD/YY), phase of the study, results as both Ct and gc/l, collection GPS timestamp is available, site ID, site name, Area and GPS. gc/l was deteremine d using serial dilutions of known copie numbers of a genomic fragement that contaims the N1 target. Interpolation was based on the log(10) of the mean of three serial dilution curves ranging from 0.001 copies/ml to 10,000 copies/ml using sigmoidal, 4PL curve ($P < 0.0021$**)
