# Supplementary material for: Utilizing river and wastewater as a SARS-CoV-2 surveillance tool in settings with limited formal sewage systems
Source: Nat Commun. 2023 Nov 30;14:7883. doi: 10.1038/s41467-023-43047-y (PMC10689440; doi:10.1038/s41467-023-43047-y)
Supplement: Supplementary file 6 — Reporting Summary [file 41467_2023_43047_MOESM6_ESM.pdf]

nature portfolio

Corresponding author(s): Barnes

Last updated by author(s): Oct 19, 2023

Reporting Summary

Nature Portfolio wishes to improve the reproducibility of the work that we publish. This form provides structure for consistency and transparency in reporting. For further information on Nature Portfolio policies, see our Editorial Policies and the Editorial Policy Checklist.

Statistics

For all statistical analyses, confirm that the following items are present in the figure legend, table legend, main text, or Methods section.

n/a	Confirmed	
☐	☒	The exact sample size (*n*) for each experimental group/condition, given as a discrete number and unit of measurement
☐	☒	A statement on whether measurements were taken from distinct samples or whether the same sample was measured repeatedly
☐	☒	The statistical test(s) used AND whether they are one- or two-sided<br>*Only common tests should be described solely by name; describe more complex techniques in the Methods section.*
☐	☒	A description of all covariates tested
☐	☒	A description of any assumptions or corrections, such as tests of normality and adjustment for multiple comparisons
☐	☒	A full description of the statistical parameters including central tendency (e.g. means) or other basic estimates (e.g. regression coefficient) AND variation (e.g. standard deviation) or associated estimates of uncertainty (e.g. confidence intervals)
☐	☒	For null hypothesis testing, the test statistic (e.g. *F*, *t*, *r*) with confidence intervals, effect sizes, degrees of freedom and *P* value noted<br>*Give P values as exact values whenever suitable.*
☐	☒	For Bayesian analysis, information on the choice of priors and Markov chain Monte Carlo settings
☒	☐	For hierarchical and complex designs, identification of the appropriate level for tests and full reporting of outcomes
☐	☒	Estimates of effect sizes (e.g. Cohen's *d*, Pearson's *r*), indicating how they were calculated

Our web collection on statistics for biologists contains articles on many of the points above.

Software and code

Policy information about availability of computer code

Data collection	KoBoToolBox (https://kf.KoBoToolbox.org/)
Data analysis	BEAST (v1.10.4), FREYJA (v1.3.10), Guppy (v5.0.7) Binomial GLM (Quasi Binomial), multi-variant normal distribution fit, minimap2 (v2.24), gofasta (v1.1.0), IQ-TREE2(v 2.2.0.3)

For manuscripts utilizing custom algorithms or software that are central to the research but not yet described in published literature, software must be made available to editors and reviewers. We strongly encourage code deposition in a community repository (e.g. GitHub). See the Nature Portfolio guidelines for submitting code & software for further information.

Data

Policy information about availability of data

All manuscripts must include a data availability statement. This statement should provide the following information, where applicable:

- Accession codes, unique identifiers, or web links for publicly available datasets
- A description of any restrictions on data availability
- For clinical datasets or third party data, please ensure that the statement adheres to our policy

The SARS-CoV-2 genomes generated in this study have been deposited in the NCBI Sequence Read Archive under BioProject ID PRJNA887942: PATH ES of SARS-CoV-2 -MLW (https://www.ncbi.nlm.nih.gov/bioproject/?term=PRJNA887942). The raw including dates, GPS coordinates and PCR results are provided in Data File S2.

April 2023

Research involving human participants, their data, or biological material

Policy information about studies with human participants or human data. See also policy information about sex, gender (identity/presentation), and sexual orientation and race, ethnicity and racism.

Reporting on sex and gender	NA
Reporting on race, ethnicity, or other socially relevant groupings	NA
Population characteristics	NA
Recruitment	NA
Ethics oversight	Wastewater samples, although not human subjects, were collected under the ethical waiver P.07/20/3089 from the College of Medicine Research Support Centre (CoMREC). No identifiable information was used for the estimations of detection frequency between ES and the population. Nevertheless, active surveillance data was collected under CoMREC P.08/20/3099 and the Liverpool School of Tropical Medicine Research Ethics Committee (LSTMREC 21-058). Passive surveillance was collated by the District Health Office and no identifiable information was used for this analysis.

Note that full information on the approval of the study protocol must also be provided in the manuscript.

Field-specific reporting

Please select the one below that is the best fit for your research. If you are not sure, read the appropriate sections before making your selection.

☐ Life sciences ☐ Behavioural & social sciences ☒ Ecological, evolutionary & environmental sciences

For a reference copy of the document with all sections, see nature.com/documents/nr-reporting-summary-flat.pdf

Ecological, evolutionary & environmental sciences study design

All studies must disclose on these points even when the disclosure is negative.

Study description	COVID-19 has impacted health systems unequally with sustained community surveillance limited globally. Work in Malawi highlights how wastewater can be used to detect emerging waves, identify variants of concern, and provide an early warning system
Research sample	wastewater from river and defunct treatment plant. We chose 7 key collection sites and then scaled to 112 collection sites to capture >80% of the Blantyre population (~1,000,000people). Site selection was undertaken using a GIS-based framework, all river confluence points within the city were selected. In addition we collect at one defunct wasterwater treatment plant were there is a major accumulation of river and potential sewage from >50% of the city
Sampling strategy	grab sample, there was no sample size calculation. Sample size was based on number of collection points that would cover the majority of the population. We samples for 2 years to determine temporal trends verse peaks based on the importation of new variants. Total samples = 2526 unique samples
Data collection	May 2020-May 2022. A two year period was chosen to capture both trends driven by clinical peaks as well as potential temporal peaks that were not observed in this dataset. Samples from each location were collected weekly from May 14th - Dec 18th, 2020 (phase 1) with gaps in the collection largely due to safeguarding of the field team as the COVID-19 pandemic unfolded in Malawi. During Phase 2 we started collecting Jan 3rd, 2021, with full scale up by mid-February, 2021 of 112 sites collected about every 2 weeks or as needed due to changes in river dynamics and safety of the field team 40-50 sites/week
Timing and spatial scale	Weekly and bi-weekly collection using SIM enabled tablet so timestamp is available for each sample. Collection cover Blantyre Malawi
Data exclusions	No data was excluded from this analysis
Reproducibility	PCR detection of SARS-CoV-2 was performed once for all sample. For positive samples were extraction and sequenced using both MinION and Illumina platforms. For key samples (ex. Omicron SNPs) a matched banked water sample was extracted and again sequenced with Mionion and Illumina. Therefore all sequencing had 2-4 replicate sequences
Randomization	Collection sites are processed at random
Blinding	All samples are blinded

Did the study involve field work? ☒ Yes ☐ No

Field work, collection and transport

Field conditions	Urban and peri-urban rivers and defunct water treatment plan. The water samples contained fecal matter from both human and animal and covered key river confluence throughout the city of Blantyre Malawi. Rainfall and temperature did not have an effect on SARS-CoV-2 detection. No additional water purity measurements were taken
Location	Blantyre Malawi is located at 15.700-15.900°S and 34.950-35.150°E. All sites GPS is given in Data File S2
Access & import/export	All access and sample collection was covered by the ethical waiver P.07/20/3089 from the College of Medicine Research Support Centre (CoMREC). No identifiable information was used for the estimations of detection frequency between ES and the population. All samples testeing was carried out in Malawi except matched sequencing using the Illumina platform. Samples shipped to Liverpool UK were covered on an MTA linked to ethics P.07/20/3089
Disturbance	No major disturbance of river ways or roads was caused by this study. Community engagement was carried out prior to river collections to ensure understanding and acceptance of the study. At any point during the study if a community leader or chief did not want to continue having collections in their community we would remove the site from the study

Reporting for specific materials, systems and methods

We require information from authors about some types of materials, experimental systems and methods used in many studies. Here, indicate whether each material, system or method listed is relevant to your study. If you are not sure if a list item applies to your research, read the appropriate section before selecting a response.

Materials & experimental systems

n/a	Involved in the study
☒	☐ Antibodies
☒	☐ Eukaryotic cell lines
☒	☐ Palaeontology and archaeology
☒	☐ Animals and other organisms
☒	☐ Clinical data
☒	☐ Dual use research of concern
☒	☐ Plants

Methods

n/a	Involved in the study
☒	☐ ChIP-seq
☒	☐ Flow cytometry
☒	☐ MRI-based neuroimaging
